# Association between sleep habits and behavioral problems in early adolescence: a descriptive study

**DOI:** 10.1186/s40359-022-00958-7

**Published:** 2022-11-05

**Authors:** Rikuya Hosokawa, Riho Tomozawa, Megumi Fujimoto, Sumire Anzai, Mai Sato, Haruko Tazoe, Toshiki Katsura

**Affiliations:** 1 Department of Human Health Sciences, Graduate School of Medicine, Kyoto University, Kyoto, 606-8507 Japan; 2 Osaka Seikei University, Osaka, 533-0007 Japan; 3 Faculty of Nursing, Meiji University of Integrative Medicine, Kyoto, 629-0392 Japan

**Keywords:** Early adolescence, Sleep habits, Behavioral problems, Japan

## Abstract

**Background:**

Sleep habits are related to children's behavior, emotions, and cognitive functioning. A strong relationship exists between sleep habits and behavioral problems. However, precisely which sleep habits are associated with behavioral problems remains unclear. Therefore, the purpose of this study is to clarify the relationship between sleep habits and behavioral problems in early adolescence.

**Methods:**

This study used data from a larger longitudinal research, specifically, data from the year 2021. First-year junior high school students (12–14 years) in Japan were surveyed; their parents (N = 1288) completed a parent-report questionnaire. The main survey items were subject attributes, the Pittsburgh Sleep Quality Index (PSQI), and the Strength and Difficulties Questionnaire (SDQ).

**Results:**

Of the 652 valid responses received, 604 individuals who met the eligibility criteria (no developmental disability in the child and completion of all survey items) were included in the analysis. To examine the relationship between sleep habits and behavioral problems, logistic regression analysis using the inverse weighted method with propensity score was conducted with sleep habits (sleep quality, time to fall asleep, sleep duration, sleep efficiency, sleep difficulty, use of sleeping pills, difficulty waking during the day, and sleep disturbances) as explanatory variables and behavioral problems (overall difficulty in SDQ) as objective variables. The propensity score was calculated by employing the logistic regression using the inverse weighted method based on propensity scores. Propensity scores were calculated based on gender, family structure, household income, and parental educational background. The results showed that behavioral problems tended to be significantly higher in the group at risk for sleep quality, sleep difficulties, daytime arousal difficulties, and sleep disturbances than in the group with no risk.

**Conclusion:**

The results suggest that deterioration in sleep quality, sleep difficulties, daytime arousal difficulties, and sleep disturbances may increase the risk of behavioral problems in adolescents.

## Background

Sleep is a biological process necessary for survival and critical to healthy living [1]. It plays an important role in brain function and impacts systemic physiology, metabolism, appetite regulation, immune system functioning, and hormonal and cardiovascular system function [2]. A key component of a healthy lifestyle is adequate sleep at appropriate times, devoid of sleep disturbances [3–5]. Sleep duration, quality of sleep, sleep timing and regularity, and absence of sleep disturbances or disorders influence overall sleep quality [6]. However, a significant proportion of the population (approximately 20%) experiences sleep deprivation or poor-quality sleep [7–10]. Sleep problems refer to deficiencies in the quantity and quality of sleep. Issues that affect the continuity of sleep are collectively referred to as sleep disorders.

Sleep disorders are characterized by persistent sleep disturbances and associated daytime dysfunction [11]. Numerous factors can cause sleep disturbances, ranging from lifestyle and environmental factors to sleep disorders and other medical conditions. Sleep disturbances have significant negative short- and long-term health consequences. Poor sleep quality could be a precursor to chronic diseases, such as obesity, cardiovascular disease, diabetes, and psychopathology [12–17]. Chronic sleep issues and wakefulness disorders adversely affect physical and mental health as well as work, academic, and driving activities [18–21]. Studies suggest that chronic insomnia is a risk factor for other mental health issues [22, 23].

Sleep problems are common across developmental stages. Numerous studies have shown that children who do not get enough sleep are more likely to develop behavioral problems. Prior research has found that impaired sleep in children is strongly associated with stress, aggression, delinquency, attention problems, and poor concentration [24]. In addition, sleep supports physical and neurobiological development as well as emotional growth; it promotes academic learning, academic performance, and cognitive functioning [25–28]. Furthermore, many studies have reported that lesser sleep duration and sleep problems are associated with lower academic performance, poor school adjustment, and psychopathology [29–31]. Sleep disorders also increase the risk of developing and exacerbating symptoms of obesity, lifestyle-related diseases, and depression. Current research in this area has investigated the relationship between sleep and proper cognitive and psychological functioning. Sleep disturbances have been associated with behavioral and emotional problems in children [32, 33]. Studies examining the association between sleep quality and behavioral problems in children have demonstrated a link between children’s externalizing and internalizing problems and poor sleep quality [34–36]. Furthermore, a robust relationship has been noted between sleep problems and increased aggression and behavioral problems among children [37, 38]. However, the relationship between sleep habits and behavioral problems in adolescents is not fully understood.

Late bedtimes and inadequate sleep, that is, short sleep, disrupted sleep patterns, insomnia, and daytime sleepiness, have become increasingly common. Growing evidence confirms that sleep problems among the youth significantly hinder learning, cognition, and memory [39, 40]. Sleep problems adversely affect behavior, social competence achievement, and quality of life, and are more pervasive than educational and health professionals realize. Disruptive sleep habits can lead to excessive daytime sleepiness (EDS), which is a symptom associated with sleep disturbances [41]. Daytime sleepiness is closely linked with poor school performance and negative perceptions of quality of life. Adolescent sleep is characterized by delayed sleep timing due to a myriad of biological, psychological, and social changes, and the characteristics of the particular stage of life. This delay in sleep onset impairs adaptation to daily timelines and makes it difficult for teenagers to stay awake. This is especially true in situations where they need to be awake—for example, during school hours. For adolescents, sleep quality is especially important because it is critical for brain development and memory processes [42]. Inadequate sleep is associated with negative outcomes in several areas of health and functioning, including physical and psychosocial health, school performance, and risk avoidance behaviors.

Among adolescents, middle school students are busy with daily classes, as well as club activities and studies. As middle school demands more complex academic tasks, adolescents with chronic sleep deprivation or short sleep duration may have poorer daytime functioning and academic performance [43]. The average sleep duration for middle school students is reported to be 7 h and 40 min in the seventh grade and less than 7 h in the ninth grade. As students advance through the grades, they tend to sleep less. Evidence suggests that sleep needs change over one’s life cycle [44]. When sleep duration at night is less than 7 h, alertness and academic performance are objectively impaired, which can affect normal development and quality of life [45, 46]. In particular, first graders are at a high risk of developing sleep disturbances during their elementary school period. Moreover, behavioral and emotional problems have become a major mental health issue among the youth in recent years, with 10–20% of youth worldwide experiencing mental disorders [47]. Such problems appear at a very young age and increase significantly during adolescence. Studies have indicated that behavioral and emotional problems can lead to mood disorders and self-harm [48]. However, the relationship between sleep habits and behavioral problems among adolescents is not fully understood. Therefore, it is important to clarify the association between healthy sleep habits and behavioral problems among middle school students.

Aspects of measuring sleep habits include not only sleep duration, but also sleep quality and onset time. Sleep quality is generally defined in terms of total sleep time, latency to fall asleep, sleep efficacy, degree of fragmentation, and sleep-disturbing events [49–52]. Although various sleep measures have been developed, the most widely used is the Pittsburgh Sleep Quality Index (PSQI) [53]. The present study aims to clarify the association between sleep habits and behavioral problems in early adolescence, as this association remains unclear.

### Current study

We aimed to understand the relationship between sleep habits and behavioral problems in early adolescence.

## Methods

### Participants

This study is part of a research project examining the impact of the parenting environment on children’s social development and adaptation. For this project, we recruited five-year old children from 52 kindergartens and 78 preschools in Nagoya, Aichi, a major metropolitan area in Japan, in 2014. Since then, we have conducted periodic surveys with these participants each year.

The current study used data from the year 2021. A parent-report questionnaire was administered to a follow-up sample of parents (N = 1288) whose children are currently 12–14 years and in the first grade. The questionnaires were completed by the children’s parents (652 valid responses received). To accurately determine the association between children’s sleep habits and behavioral characteristics, children diagnosed with developmental disabilities and those who did not complete the required items of the questionnaire were excluded from the analysis of this study. As a result, 604 (92.6%) of the 652 children met the eligibility criteria. The mean age of the children was 13.12 years (standard deviation = 0.52, boys: n = 296, girls: n = 308; the mean age was calculated by dividing the age in months by one year).

### Ethics statement

At the beginning of the study, the children’s parents were informed about the purpose of the study and associated procedures, and were made aware that participation in the baseline study was voluntary. Parents provided written informed consent on behalf of their children prior to their participation in the study. Ethical approval for this study was obtained from the Kyoto University Ethics Committee (E2322).

### Measures

#### Explanatory variable: sleep condition

In this study, sleep condition was assessed with the PSQI [53], which is a global measure of sleep quality based on retrospective assessments of sleep behaviors. The PSQI is a self-administered questionnaire consisting of 18 items on sleep status during the past month. Through these 18 items, 7 components are calculated: subjective sleep quality, sleep latency, sleep duration, sleep efficiency, sleep disturbances, sleep medication use, and daytime dysfunction. Each component was rated on a score of 0–3, with a score of 1 or higher indicating being at risk and a score of 0 indicating not being at risk. The sum of these scores was calculated as the global PSQI score, ranging from 0–21. Higher scores indicate more severe sleep problems, including low sleep quality. The PSQI is used to diagnose various sleep disorders and insomnia. The Japanese version was used in this study [54]. It is a highly reliable and valid scale. The sum of the scores for the seven dimensions yields one global score. This global PSQI score has a cutoff of 5 to distinguish between “good sleepers” and “poor sleepers.” A PSQI score of > 5 indicates poor sleep quality and being at risk of sleep problems, whereas a PSQI score ≤ 5 indicates good sleep quality and having a low risk of sleep problems [55].

#### Objective variable: children’s behaviors

In this study, children’s behaviors were assessed with the Strengths and Difficulties Questionnaire (SDQ) [56]. The SDQ is a widely validated instrument for identifying behavioral and emotional problems, as well as prosocial behaviors (25 items). However, in this study, items on prosocial behaviors (5 items) were excluded. The Japanese version, which is a highly reliable and validated tool, was used in this study [57]. The SDQ consists of 20 items assessing behavioral and emotional problems; these items are rated on a three-point Likert scale. Emotional and behavioral problems include emotional symptoms, conduct problems, hyperactivity-inattention, and peer problems, which are combined into a total difficulty score by summing the scores of the first four subscales from 0 (low difficulty) to 40 (high difficulty). Higher scores on this scale indicate more emotional and behavioral problems. More specifically, the SDQ total scores are categorized as “normal” (range 0–13), “borderline” (range 14–16), and “abnormal” (range 17–40), indicating the presence of general psychopathology. Therefore, in this study, scores above 17 were considered “abnormal” and scores below 17 were considered “normal.”

### Demographic covariates

Parent-reported information was collected on the child’s gender, family structure, household income, and parental education level.

### Data analyses

The purpose of this study was to determine the association between sleep habits and behavioral problems. A propensity score method was used to address possible bias due to original sleep habits. In previous studies, sleep problems have been shown to be influenced by child demographics, socioeconomic status, and household characteristics [58]. Therefore, in this study, a propensity score was calculated using gender, family structure, household income, and parental education level as variables that could potentially influence sleep habits. To reduce the effects of bias and potential confounding effects, this study used inverse probability weighting to rigorously adjust for significant differences in participant characteristics. The inverse of the propensity score was incorporated into a weighted logistic regression model to calculate odds ratios (ORs) for behavioral problems due to sleep habits (SDQ total scores). The statistical analyses were performed using SPSS version 27.0.

## Results

Table 1 shows the participants’ demographics. The median annual income was ¥5–6 million. University or graduate school was the most common educational background for both mothers and fathers.

**Table 1:** Participant characteristics

	*N*	%
*Sex*		
Male	296	49.0
Female	308	51.0
*Family composition*		
Single-parent household	44	7.3
Two-parent household	560	92.7
*Annual household income (¥)*		
< 3,000,000	46	7.7
3,000,000–6,000,000	246	41.4
6,000,000–9,000,000	169	28.5
≥ 9,000,000	133	22.4
*Mother’s educational background*		
Middle school or high school	109	18.2
Junior college or vocational school	241	40.2
University or graduate school	249	41.6
*Father’s educational background*		
Middle school or high school	140	24.0
Junior college or vocational school	76	13.0
University or graduate school	367	63.0

Table 2 shows the participants’ quality of sleep and behavioral problems. The mean sleep duration was 464.31 (52.43) min or 7.74 h.

**Table 2:** Participants’ quality of sleep and behavioral problems

	*N*	%
*Subjective sleep quality*		
No-risk group (= 0)	182	30.9
Risk group (≥ 1)	407	69.1
*Sleep latency*		
No-risk group (= 0)	325	56.3
Risk group (≥ 1)	252	43.7
*Sleep duration*		
No-risk group (= 0)	540	91.4
Risk group (≥ 1)	51	8.6
*Habitual sleep efficiency*		
No-risk group (= 0)	567	98.1
Risk group (≥ 1)	11	1.9
*Sleep disturbances*		
No-risk group (= 0)	313	53.6
Risk group (≥ 1)	271	46.4
*Use of sleeping medication*		
No-risk group (= 0)	591	99.3
Risk group (≥ 1)	4	0.7
*Daytime dysfunction*		
No-risk group (= 0)	305	53.4
Risk group (≥ 1)	266	46.6
*PSQI-global score*		
No-risk group (≤ 5)	499	94.5
Risk group (> 5)	29	5.5
*Behavioral problems*		
No-risk group (< 17)	565	93.5
Risk group (≥ 17)	39	6.5

PSQI, Pittsburgh Sleep Quality Index; Sleep Disorder; PSQI-global score > 5; Strength and Difficulties Questionnaire; total difficulties score ≥ 17

Tables 3 and 4 shows the association between student attributes and sleep habits. Gender was not associated with sleep habits. In terms of family structure, a higher proportion of children in single-parent households were at risk of a higher PSQI-global score compared to those in two-parent households. In terms of annual household income, children from families with annual incomes of less than ¥3 million were at higher risk of poor subjective sleep quality than those from families with annual incomes of more than ¥3 million. In terms of mothers’ educational background, those whose mothers were less educated (having completed only middle/high school) were more likely to be at risk for poor subjective sleep quality than children whose mothers received higher education (junior college, vocational school, or university/graduate education). Risks of habitual sleep efficiency, sleep disturbances, and use of sleeping medication were higher for the former than the latter. Fathers’ educational background showed no association with students’ sleep habits.

**Table 3:** Participant characteristics and quality of sleep

	Subjective sleep quality					Sleep latency					Sleep duration					Habitual sleep efficiency				
	No-risk group		Risk group			No-risk group		Risk group			No-risk group		Risk group			No-risk group		Risk group		
	*n*	%	*n*	%	*p*	*n*	%	*n*	%	*p*	*n*	%	*n*	%	*p*	*n*	%	*n*	%	*p*
*Sex*																				
Male	89	30.7	201	69.3	0.913	166	59.1	115	40.9	0.195	269	92.8	21	7.2	0.238	277	97.9	6	2.1	0.708
Female	93	31.1	206	68.9		159	53.7	137	46.3		271	90.0	30	10.0		290	98.3	5	1.7	
*Family composition*																				
Single-parent household	7	17.9	32	82.1	0.070	18	42.9	24	57.1	0.068	36	90.0	4	10.0	0.749	35	94.6	2	5.4	0.107
Two-parent household	175	31.8	375	68.2		307	57.4	228	42.6		504	91.5	47	8.5		532	98.3	9	1.7	
*Annual household income (¥)*																				
< 3,000,000	5	11.6	38	88.4	**0.029**	18	41.9	25	58.1	0.281	40	87.0	6	13.0	0.642	42	97.7	1	2.3	0.150
3,000,000–6,000,000	81	33.8	159	66.3		134	57.3	100	42.7		222	92.1	19	7.9		229	98.3	4	1.7	
6,000,000–9,000,000	50	30.3	115	69.7		93	57.4	69	42.6		151	92.1	13	7.9		156	96.3	6	3.7	
≥ 9,000,000	45	34.4	86	65.6		73	57.0	55	43.0		117	90.0	13	10.0		130	100.0	0	0.0	
*Mother’s educational background*																				
Middle school or high school	27	25.5	79	74.5	0.331	48	46.6	55	53.4	0.088	98	93.3	7	6.7	0.322	92	94.8	5	5.2	**0.039**
Junior college or vocational school	73	31.3	160	68.7		134	59.0	93	41.0		210	89.4	25	10.6		228	98.7	3	1.3	
University or graduate school	82	33.5	163	66.5		140	57.9	102	42.1		228	92.7	18	7.3		242	98.8	3	1.2	
*Father’s educational background*																				
Middle school or high school	43	32.1	91	67.9	0.812	73	57.0	55	43.0	0.970	120	90.2	13	9.8	0.763	123	97.6	3	2.4	0.447
Junior college or vocational school	21	28.4	53	71.6		42	58.3	30	41.7		69	92.0	6	8.0		72	100.0	0	0.0	
University or graduate school	116	32.1	245	67.9		202	56.7	154	43.3		334	92.3	28	7.7		352	98.1	7	1.9	

χ^2^ analysis. Bold numbers indicate statistically significant differences

**Table 4:** Participant characteristics and quality of sleep

	Sleep disturbances					Use of sleeping medication					Daytime dysfunction					PSQI-global score				
	No-risk group		Risk group			No-risk group		Risk group			No-risk group		Risk group			No-risk group		Risk group		
	*n*	%	*n*	%	*p*	*n*	%	*n*	%	*p*	*n*	%	*n*	%	*p*	*n*	%	*n*	%	*p*
*Sex*																				
Male	153	53.5	133	46.5	0.962	289	99.0	3	1.0	0.298	153	54.4	128	45.6	0.626	248	95.0	13	5.0	0.610
Female	160	53.7	138	46.3		302	99.7	1	0.3		152	52.4	138	47.6		251	94.0	16	6.0	
*Family composition*																				
Single-parent household	20	48.8	21	51.2	0.521	41	97.6	1	2.4	0.160	19	48.7	20	51.3	0.542	27	84.4	5	15.6	**0.009**
Two-parent household	293	54.0	250	46.0		550	99.5	3	0.5		286	53.8	246	46.2		472	95.2	24	4.8	
*Annual household income (¥)*																				
< 3,000,000	18	40.9	26	59.1	0.116	43	97.7	1	2.3	0.187	21	48.8	22	51.2	0.329	30	85.7	5	14.3	0.098
3,000,000–6,000,000	132	56.7	101	43.3		239	98.8	3	1.2		114	49.8	115	50.2		200	93.9	13	6.1	
6,000,000–9,000,000	81	49.1	84	50.9		166	100.0	0	0.0		94	58.8	66	41.3		141	95.9	6	4.1	
≥ 9,000,000	76	57.6	56	42.4		133	100.0	0	0.0		70	54.3	59	45.7		118	95.9	5	4.1	
*Mother’s educational background*																				
Middle school or high school	47	46.1	55	53.9	**0.047**	104	97.2	3	2.8	**0.011**	47	46.1	55	53.9	0.153	77	89.5	9	10.5	0.091
Junior college or vocational school	139	59.4	95	40.6		237	100.0	0	0.0		118	52.4	107	47.6		197	95.6	9	4.4	
University or graduate school	124	51.0	119	49.0		245	99.6	1	0.4		137	57.3	102	42.7		220	95.2	11	4.8	
*Father’s educational background*																				
Middle school or high school	77	58.3	55	41.7	0.441	134	98.5	2	1.5	0.073	64	49.2	66	50.8	0.394	106	93.0	8	7.0	0.492
Junior college or vocational school	36	49.3	37	50.7		73	98.6	1	1.4		37	51.4	35	48.6		63	95.5	3	4.5	
University or graduate school	193	53.8	166	46.2		364	100.0	0	0.0		195	55.9	154	44.1		316	95.8	14	4.2	

*χ*^2^ analysis. Bold numbers indicate statistically significant differences

Table 5 shows the association between sleep habits and behavioral problems. To examine this association, logistic regression analysis using the inverse weighted method with propensity score matching was conducted, with the explanatory variables being sleep habits (sleep quality, time to fall asleep, sleep duration, sleep efficiency, sleep difficulty, use of sleeping pills, difficulty waking during the day, and sleep disturbances) and the objective variables being behavioral problems (overall difficulty on the SDQ). The results showed that sleep quality (OR) was significantly higher than that of the other variables. Additionally, behavioral problems were significantly higher in the group at risk for poor sleep quality, sleep difficulties, daytime arousal difficulties, and sleep disturbances than in the group with no risk.

**Table 5:** Association between quality of sleep and behavioral problems

	Crude model				IPTW model			
	OR	95% CI		*p*	OR	95% CI		*p*
*Subjective sleep quality*								
No-risk group (= 0)	Ref				Ref			
Risk group (≥ 1)	4.000	1.395	11.467	**0.010**	3.373	1.159	9.811	**0.026**
*Sleep latency*								
No-risk group (= 0)	Ref				Ref			
Risk group (≥ 1)	1.930	0.991	3.760	0.053	1.993	0.974	4.078	0.059
*Sleep duration*								
No-risk group (= 0)	Ref				Ref			
Risk group (≥ 1)	1.657	0.616	4.453	0.317	1.785	0.593	5.369	0.302
*Habitual sleep efficiency*								
No-risk group (= 0)	Ref				Ref			
Risk group (≥ 1)	5.713	1.450	22.518	**0.013**	2.834	0.628	12.802	0.176
*Sleep disturbances*								
No-risk group (= 0)	Ref				Ref			
Risk group (≥ 1)	3.035	1.475	6.245	**0.003**	2.473	1.165	5.251	**0.018**
*Use of sleeping medication*								
No-risk group (= 0)	Ref				Ref			
Risk group (≥ 1)	4.991	0.506	49.194	0.169	3.452	0.350	34.023	0.289
*Daytime dysfunction*								
No-risk group (= 0)	Ref				Ref			
Risk group (≥ 1)	13.040	3.930	43.265	** < 0.001**	11.295	3.345	38.139	** < 0.001**
*PSQI-global score*								
No-risk group (≤ 5)	Ref				Ref			
Risk group (> 5)	9.577	3.906	23.480	** < 0.001**	8.988	3.119	25.898	** < 0.001**

Bold numbers indicate statistically significant differences

IPTW model, inverse probability of treatment weighted model; OR, odds ratio; CI, confidence interval; PSQI, Pittsburgh Sleep Quality Index

## Discussion

The mean sleep duration was found to be 464.31 min or 7.74 h. Gender was not associated with sleep habits. A higher proportion of children in single-parent households were at risk of a higher PSQI-global score. Children from families with annual incomes of less than ¥3 million were at higher risk of poor subjective sleep quality. Those whose mothers were less educated (having completed only middle/high school) were more likely to be at risk for poor subjective sleep quality. Fathers’ educational background showed no association with students’ sleep habits. Behavioral problems were found to be significantly higher in the group at risk for poor sleep quality, sleep difficulties, daytime arousal difficulties, and sleep disturbances than in the group with no risk. These results are consistent with those of previous studies, indicating that sleep problems are common in adolescents and can affect behavioral and emotional functioning [24, 59–63].

In this study, the association between daytime sleepiness and behavioral problems was particularly strong. Sleep habits have a direct influence on daytime activity. Poor sleep habits can lead to EDS, one of the main symptoms associated with sleep disturbances. Excessive sleepiness, defined as sleepiness that occurs under conditions in which one would normally expect to be awake, is characterized by EDS, which in turn is caused by a variety of sleep disorders and adversely affects academic performance [64–67]. Untreated sleep disturbances and associated EDS can lead to behavioral problems, mood disorders, depression, emotional dysregulation, and neurocognitive dysfunction [17, 68–71]. EDS in children and adolescents is underreported by parents and underdiagnosed by physicians [72]. Prompt detection, diagnosis, and management of EDS are essential.

The average sleep duration for participants in this study was 464.31 (± 52.43) min, or 7 h and 44 min, similar to the average sleep duration in Japan [73]. However, according to the Organisation for Economic Development and Co-operation, Japanese people sleep less than people in the rest of the world [9]. Japan ranks second among countries with the shortest sleep duration (7 h 41 min), compared with France (8 h 48 min), which has the longest sleep duration among developed countries [74]. Adolescents aged 14–17 years need 8–10 h of sleep per day [75]. However, it is well known that children and adolescents get less than the recommended hours of sleep [76, 77]. This pattern of sleep deprivation in children and adolescents is thought to be due to a combination of both intrinsic and extrinsic factors. The intrinsic factors include circadian rhythm shifts and sleep-disorder breathing during adolescence [78–80]. By contrast, the extrinsic factors, such as earlier school timings, caffeine intake, and exposure to computer games and electronic media, have also been shown to negatively affect sleep duration [81–87]. Middle school requires more complex academic tasks than elementary school, and adolescents with chronic sleep deprivation or short sleep duration may have poorer daytime functioning and academic performance. Several studies on the neural effects of sleep restriction in early adolescence support the idea that critical prefrontal development at this stage of life has serious behavioral consequences in adulthood [88–90]. Thus, the current sleep duration and sleep quality of the youth needs to be increased.

It is important to intervene as early as possible to ensure that persistent sleep problems are not strongly associated with other behavioral problems, such as anxiety, depression, or aggression. Behavioral therapeutic approaches for children with bedtime problems and nighttime awakenings are supported in the literature and should be the first treatment option [91].

Interventions to improve sleep habits are possible. It is strongly recommended that sleep problems be considered in comprehensive assessments, especially for children with psychiatric problems, and that preventive education be part of the usual standard of care. Prior studies have confirmed the effectiveness of behavioral therapy for bedtime problems and frequent nighttime awakening. Evidence has been presented on the efficacy of behavioral therapy in treating people with insomnia [92]. Additionally, behavioral interventions, such as establishing routines and preventive education for parents, have been identified as effective treatments to improve sleep patterns [33, 93–95]. Thus, treating insomnia and improving sleep quality may be beneficial for improving children’s learning, aggression, mood, and behavior. In addition and importantly, research is required to assess the impact of interventions on children’s behavior and mood and the use of objective measures in assessing sleep patterns for appropriate interventions [93, 95, 96]. Short sleep duration and frequent nighttime awakenings at 18 months are significant predictors of the development of emotional and behavioral problems five years later [97]. Further, young children who do not sleep well on a regular basis are more likely to experience aggression and attention problems in adolescence [37]. Thus, it may be important to intervene from an earlier age. However, several studies suggest that sleep disorders may be undiagnosed in pediatric clinics [18, 98]. If sleep disorders go undiagnosed, the negative impact on daytime functioning may be significant [64, 98–101]. Thus, it may be useful to assess and provide timely intervention for adolescents with sleep problems. In particular, regulating sleep quality, sleep difficulties, and daytime arousal difficulties, as indicated in this study, may be effective in reducing the risk of behavioral problems.

## Limitations

This study has some limitations. As this was a cross-sectional study, causality could not be determined. Children with more behavioral problems may sleep less and have poorer sleep quality than those who do not show signs of behavioral problems. In addition, the PSQI and SDQ were parent-reported and, thus, vulnerable to reporting bias.

## Conclusion

The results suggest that disrupted sleep habits may increase the risk of behavioral problems. Regulating sleep quality, sleep difficulties, and daytime arousal difficulties may reduce the risk of behavioral problems in early adolescence.

## Data Availability

The data supporting the findings of this study are available from the Nagoya child care study but restrictions apply to the availability of these data, which were used under license for the current study and are thus not publicly available. Data are however available from the authors upon reasonable request and with the permission of the researchers (Rikuya Hosokawa).

## Abbreviations

PSQI: Pittsburgh Sleep Quality Index
SDQ: Strengths and Difficulties Questionnaire
EDS: Excessive daytime sleepiness
